# Comparative Evaluation of Sagittal Alignment in Total Knee Arthroplasty: Robot Sensor Versus Surgeon’s Eye and Influencing Factors

**DOI:** 10.3390/jcm14228242

**Published:** 2025-11-20

**Authors:** Dong Nyoung Lee, Chang Hyun Nam, Ji-Hoon Baek, Suengryol Ryu, Juneyoung Heo, Ji Hyun Kim, Su Chan Lee, Sang Won Lee

**Affiliations:** 1 Joint & Arthritis Research, Department of Orthopaedic Surgery, Himchan Hospital, Seoul 07999, Republic of Korea; fretless@hanmail.net (D.N.L.); changcape@naver.com (C.H.N.); jihoon011@naver.com (J.-H.B.); oszzang102@naver.com (S.R.); juneyoungheo@gmail.com (J.H.); tttis5555@gmail.com (J.H.K.); himchanhospital@naver.com (S.C.L.); 2 Harvard Combined Orthopaedic Residency Program, 55 Fruit Street, Boston, MA 02114, USA

**Keywords:** body mass index, flexion contracture, hyperextension, robot-assisted total knee arthroplasty, visual assessment

## Abstract

**Background**: Postoperative sagittal imbalance, including hyperextension and flexion contracture after total knee arthroplasty (TKA), adversely affects long-term outcomes. Conventional techniques depend on surgeons’ visual estimation, which may vary with patient anatomy. This study compared the accuracy of sagittal alignment assessment between a robot-assisted system and surgeons’ visual evaluation and analyzed the influence of body mass index (BMI) and anatomic factors on concordance. **Methods**: Sixty unilateral TKAs performed between October 2023 and May 2024 using the MAKO system were retrospectively reviewed. Sagittal mechanical axis angles were obtained from the robotic system (M group) and visually estimated by two blinded orthopedic surgeons from intraoperative lateral photographs (S group) for 9, 10, and 11 mm trial inserts. Final alignment was verified by C-arm radiographs. Inter-observer reliability was analyzed using Cohen’s κ and ICC, and correlations with BMI, thigh circumference, and limb proportions were assessed. **Results**: In patients with overweight/obesity, the S group significantly underestimated sagittal alignment (*p* < 0.001), whereas the M group maintained consistent accuracy regardless of BMI. With thinner inserts (9 mm), hyperextension was often overestimated (mismatch 55%, *p* < 0.0001), and with thicker inserts (11 mm), flexion contracture was underestimated (mismatch 46.7%, *p* = 0.001). Inter-observer reliability was good (κ = 0.717, ICC = 0.816). **Conclusions**: Visual assessment may underestimate sagittal alignment, especially in patients with obesity or those with abundant soft tissue. The MAKO robotic system provided consistent, objective alignment evaluation regardless of body habitus. Robotic-assisted quantitative assessment enables more accurate and reproducible sagittal alignment, supporting safer and more reliable TKA outcomes.

## 1. Introduction

In South Korea, knee osteoarthritis is highly prevalent, with a reported rate of 33.3%. From 2010 to 2020, a cumulative total of 622,405 primary total knee arthroplasties (TKAs) were performed for end-stage knee osteoarthritis [1]. TKA is an effective surgical intervention for the treatment of end-stage of the knee as it can decrease pain and improve function [2]. However, postoperative instability following TKA is known to negatively affect outcomes [3]. In particular, flexion contracture, one form of instability occurring in the sagittal plane, arises when the extension gap is tighter than the flexion gap during surgery. This may result from insufficient release of the posterior capsule, failure to remove posterior osteophytes, or insufficient distal femoral resection. In contrast, hyperextension develops when the extension gap is more lax than the flexion gap, which may arise from excessive relaxation of posterior structures or over-resection of the distal femur. Hyperextension and flexion contracture can lead to numerous long term clinical sequelae including decreased durability, knee instability, gait disturbance, and increased fall risk [4,5]. Thus, accurate intraoperative evaluation and prevention of these conditions are critically important.

Therefore, recently, robot-assisted surgery has been introduced to improve the precision of bone cuts and implant placement. Conventional TKA methods rely on the surgeon’s experience, leading to differences in alignment accuracy and, consequently, differences in clinical outcomes [6]. The robot-assisted surgical systems introduced so far have various platform-specific features. The ROSA Robotic System (Zimmer Biomet, Montreal, QC, Canada) performs bone cuts using imaging and sensor feedback. In terms of resection accuracy, it has demonstrated cutting errors of less than 0.6° in both the coronal and sagittal planes [7]. A semiautonomous, imageless, handheld robotic-assisted system (NAVIO Surgical System, Smith & Nephew, Memphis, TN, USA) has also shown improvements in alignment accuracy [8]. The MAKO robotic system (version 3.11, Stryker Orthopaedics, Mahwah, NJ, USA), which uses CT-based robotic arm-assisted platform for bone cutting, provides real-time monitoring of joint gaps and alignment during surgery. This approach has demonstrated improvements in the precision of alignment and gap as well as functional outcomes [9]. However, its role in preventing sagittal plane hyperextension remains unestablished.

Therefore, this study aimed to compare the accuracy of sagittal plane alignment assessment (hyperextension and flexion contracture) during TKA between the MAKO robotic-assisted system and two experienced orthopedic surgeons’ visual assessment. In addition, the study investigated the effects of various anatomic factors, including factors that may affect the identification of bony landmarks for determining the sagittal mechanical axis on lateral views (body mass index [BMI], thigh circumference) and factors associated with reduced visual sensitivity to angular changes due to longer lever arms (tibia-to-femur ratio, leg-to-body ratio) on the concordance of the results. Through this analysis, the study seeks to evaluate the clinical usefulness and reliability of robotic surgery in situations where surgical visibility is limited or where changes in soft-tissue tension are critical.

## 2. Materials and Methods

### 2.1. Ethics Statement

The design and protocol of this retrospective study were approved by the Institutional Review Board (IRB) of our hospital (IRB number: 116655-01-202310-01). The requirement for informed consent was waived because of the retrospective nature of the study.

This study retrospectively analyzed 60 TKAs cases between October 2023 and May 2024. All patients underwent history taking, physical examination, and radiographic evaluation including preoperative standing AP/lateral radiographs, intraoperative lateral radiographs, and postoperative knee AP/lateral radiographs (at 2 and 4 weeks). All surgeries were performed using the MAKO robotic-assisted system.

Inclusion criteria included patients undergoing unilateral total knee replacement and with Kellgren-Lawrence grade 3–4 end-stage osteoarthritis on radiographs. Exclusion criteria included (1) active infection; (2) intra-articular or extra-articular deformity precluding reliable visual assessment of sagittal alignment; (3) neurologic issues, including significant cognitive impairment and paralysis, or significant comorbidities, including heart failure and severe pulmonary disease, limiting postoperative rehabilitation; (4) severe spinal deformity; and (5) history of alcoholism or drug abuse.

**Figure 1:**
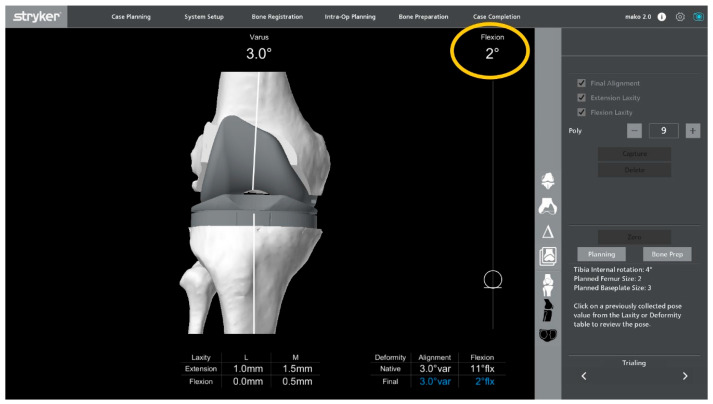
Intraoperative screenshot from the MAKO robotic system showing sagittal alignment analysis after trial insert placement.

**Figure 2:**
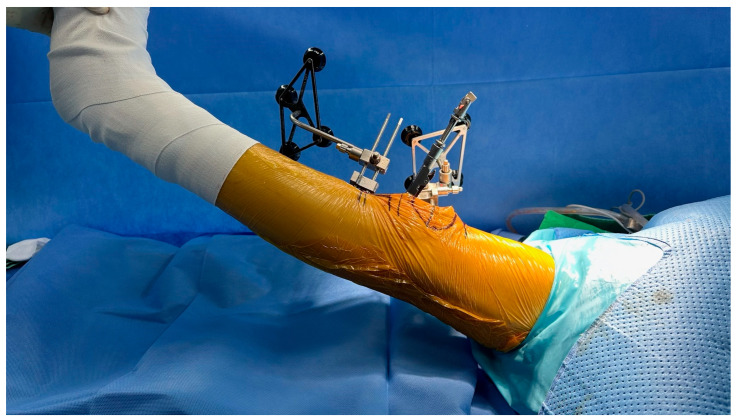
Intraoperative image showing the surgeon’s visual estimation of the sagittal mechanical axis.

Patients underwent total knee arthroplasty using the MAKO robotic system. Bone was resected with the target joint gap space set at 18 mm. After bone resection, Trial inserts of 9 mm, 10 mm, and 11 mm were sequentially inserted. The MAKO system on monitor was used to measure sagittal alignment—hyperextension or flexion contracture angles (M group, Figure 1). For comparison, total of 180 intraoperative photographs obtained from 60 TKAs, with three images captured for each case corresponding to 9 mm, 10 mm, and 11 mm trial inserts, were independently reviewed by two experienced orthopedic surgeons, who visually evaluated the sagittal mechanical axis angle and the two surgeons not involved in the TKA procedure. This assessment was designated as the Surgeon-eye Group (S group, Figure 2). The two evaluators were blinded to each other’s assessments and to the MAKO measurements, and each evaluation was recorded independently.

The primary analysis was based on the first evaluator’s results. Inter-observer agreement was assessed using Cohen’s kappa (κ) and the intra-class correlation coefficient (ICC), presented as secondary analyses.

The visually estimated sagittal alignment was compared with the measurement based on MAKO system’s haptic sensors.

**Figure 3:**
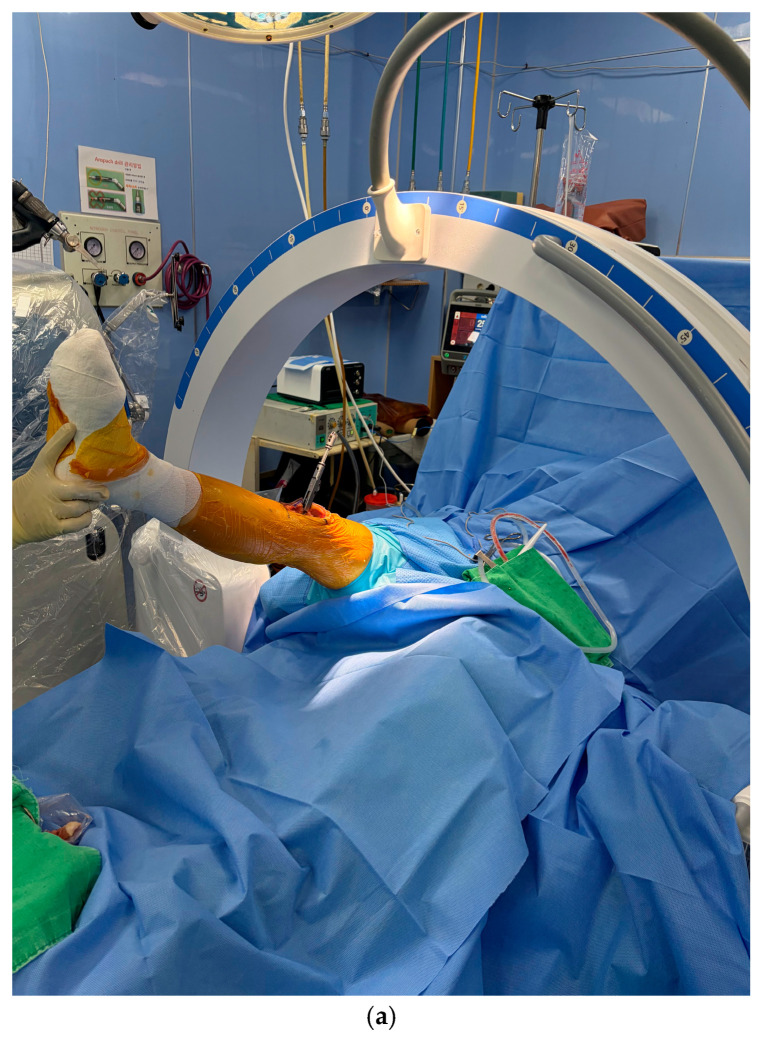

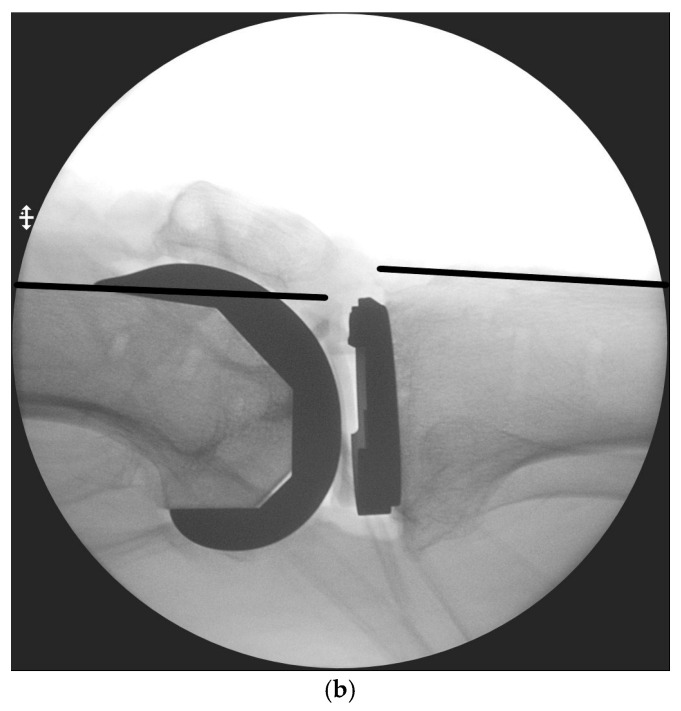
(**a**) Intraperative C-arm radiographs obtained with the final insert and C-arm beam aligned parallel to the tibial plateau. (**b**) Lateral fluoroscopic image to assess sagittal alignment intraoperatively. The angle between the anterior cortex of the distal femur and the anterior cortex of the proximal tibia is measured.

In addition, in order to evaluate how accurately the angles calculated by the MAKO robotic system reflect the actual hyperextension and flexion contracture, Intraoperative C-arm radiographs were obtained with the final insert in place after achieving ligamentous balance in the true lateral position with the knee fully extended and the C-arm beam aligned parallel to the tibial plateau (Figure 3a). The reference angle was determined by the angle between the anterior cortices of the distal femur and proximal tibia was measured to determine sagittal mechanical alignment (Figure 3b) [10].

**Figure 4:**
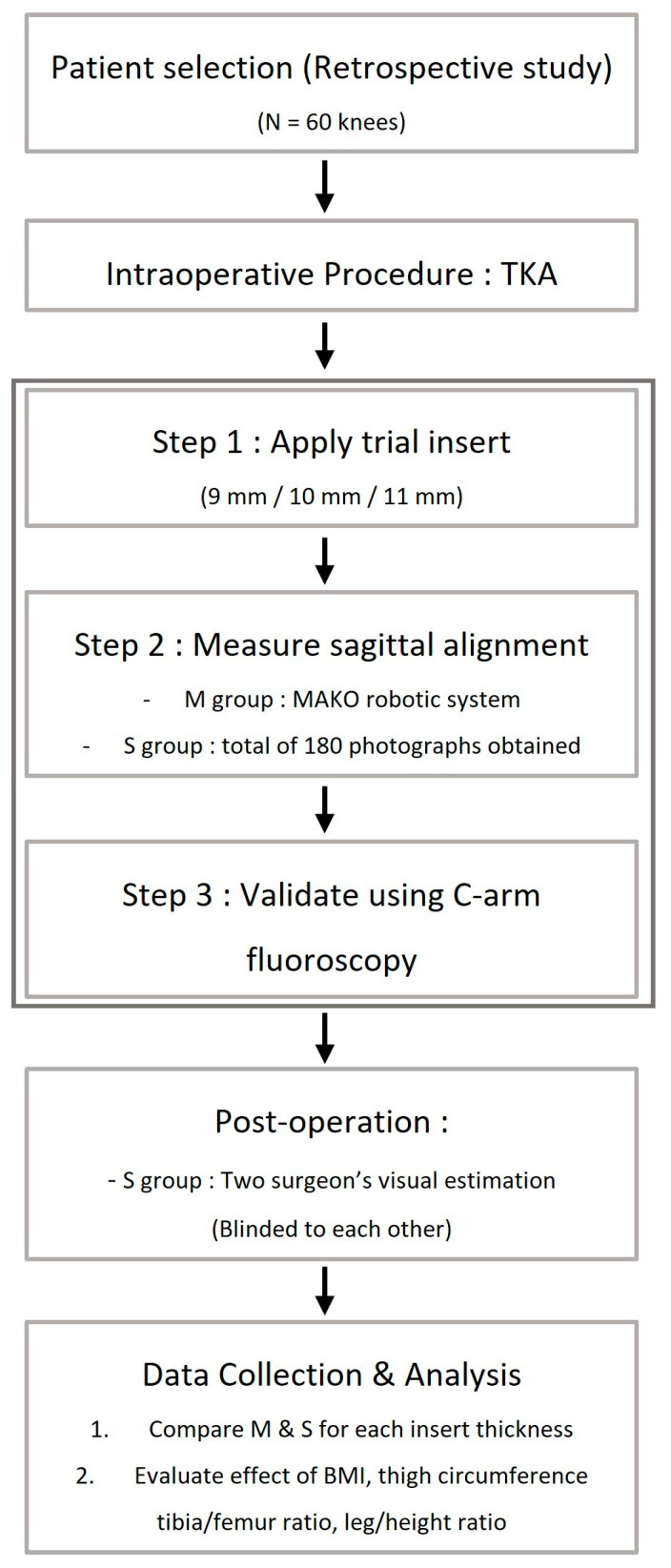
Graphical schematic of the study design and workflow.

Furthermore, patient factors that might influence a surgeon’s visual assessment of the sagittal plane alignment in the operating room, including BMI, thigh circumference, tibia-to-femur ratio, and leg-to-body ratio, were recorded and analyzed for correlation with measured sagittal plane angles. All values were based on preoperative physical measurements and radiographic data. Radiographic measurements were conducted by a blinded independent researcher (Figure 4).

### 2.2. Statistical Analysis

All statistical analyses were performed using SPSS version 27.0 (IBM Corp., Armonk, NY, USA).

Continuous variables were expressed as mean ± standard deviation (SD), and categorical variables as frequencies and percentages.

Categorical variables were compared using the chi-square test or Fisher’s exact test.

Inter-observer reliability was evaluated using Cohen’s kappa (κ) for categorical variables and the ICC (two-way random-effects model, absolute agreement) for continuous variables.

Statistical significance was set at a *p*-value of <0.05.

## 3. Results

No patients in the study experienced serious complications that could have influenced the results.

In the baseline analysis, no significant differences were observed between the normal-weight group (BMI < 25 kg/m^2^) and the overweight/obese group (BMI ≥ 25 kg/m^2^) in age (73.1 ± 5.8 vs. 72.4 ± 8.0 years, *p* = 0.676), preoperative hip–knee–ankle (HKA) angle (8.1° ± 3.3° vs. 8.5° ± 3.4°, *p* = 0.668), femur-to-tibia length ratio (0.4 ± 0.2, *p* = 0.186), or height-to-leg length ratio (0.2 ± 0.0, *p* = 0.182).

However, compared with the normal-weight group, the overweight/obese group demonstrated significantly greater BMI (28.8 ± 1.6 vs. 22.9 ± 1.3 kg/m^2^, *p* < 0.0001) and thigh circumference (48.5 ± 4.2 vs. 45.4 ± 3.3 cm, *p* = 0.002) (Table 1).

Knee hyperextension/flexion contracture angles were compared between the normal-weight and overweight/obese groups using C-arm radiographs, the MAKO robotic system (M group), and surgeon visual assessment (S group), with sagittal mechanical axis angles analyzed according to BMI.

Sagittal alignment axis using c-arm showed a mean angle of 3.4° (±3.4°) in the normal-weight group and 2.6° (±2.7°) in the overweight/obese group, with no significant difference (*p* = 0.095). In contrast, the S group showed mean angles of 2.0° (±2.7°) in the normal-weight group and 0.1° (±2.7°) in the overweight/obese group, indicating a statistically significant underestimation of sagittal mechanical axis alignment in the overweight/obese group (*p* < 0.0001). The M group showed minimal differences in mean angles between the groups (consistently around 3°), demonstrating measurement consistency regardless of BMI (Table 2).

The final real insert TKA using the MAKO robotic-assisted system averaged 10.22 mm and included 9 mm (34%), 10 mm (20%), 11 mm (36%), and 12 mm (10%).

For 9 mm, 10 mm, and 11 mm inserts, we classified the sagittal plane alignment in the M group and the S group as 0°, greater than 0° (flexion contracture), and less than 0° (hyperextension), and assessed the concordance between the two groups. When different insert thicknesses were applied in the same patient, there were clear differences in concordance of sagittal plane alignment between the M and S groups (Table 3).

Using a 9 mm insert, 34/60 (56.7%) patients in the S group were classified as neutral, 7/60 (11.7%) as flexion contracture, and 19/60 (31.7%) as hyperextension, whereas the distribution of the M group was 7/60 (11.7%), 35/60 (58.3%), and 18/60 (30.0%), respectively. The two groups were concordant in 45.0% of cases, and the difference was statistically significant (*p* < 0.0001). Using a 10 mm insert, 12/60 (20.0%) patients in the S group were classified as neutral, 34/60 (56.7%) as flexion contracture, and 14/60 (23.3%) as hyperextension, while the distribution of the M group was 10/60 (16.7%), 45/60 (75.0%), and 5/60 (8.3%), respectively. The concordance rate was 63.3%, and the difference was marginally statistically significant (*p* = 0.05). Using an 11 mm insert, the S group was classified as neutral in 12/60 (20.0%) cases, flexion contracture in 42/60 (70.0%), and hyperextension in 6/60 (10.0%), whereas the M group distribution was 0/60 (0%), 55/60 (91.7%), and 5/60 (8.3%), respectively. The concordance rate was highest at 75.0% of cases, and the difference was statistically significant (*p* < 0.0001).

In the M group, the proportion of patients with a flexion contracture increased with increasing insert thickness (58.3% → 75.0% → 91.7%), and in the S group, the proportion of patients with hyperextension decreased correspondingly (31.7% → 23.3% → 10.0%). As a result, the concordance rate between the M and S groups increased with increasing insert thickness (45.0% → 63.3% → 75.0%).

Notably, with the 9 mm insert, the S group showed a significant tendency to misclassify flexion contracture as hyperextension relative to the M method (mismatch 55.0%, *p* < 0.0001). This was highlighted by the fact that flexion contracture, defined as angles > 0°, was identified in 58.3% of cases by the M group but in only 11.7% by the S group.

For the 9, 10, and 11 mm trial inserts, sagittal alignment was classified by both the M group and S group into three categories: mild flexion contracture (0–5°), severe flexion contracture (≥6°), and hyperextension (<0°). Concordance between the two methods was then evaluated according to the degree of flexion contracture (Table 4).

With increasing insert thickness, the proportion of knees classified as severe flexion contracture (≥6°) in the M group increased progressively (8.3% → 30.0% → 61.7%). In contrast, the S group showed consistently low rates, with a marked increase observed only at 11 mm (0.0% → 1.1% → 28.3%). In the S group, the proportion classified as hyperextension decreased markedly with increasing insert thickness (31.7% → 23.3% → 10.0%), whereas in the M group, it remained relatively stable (30.0% → 8.3% → 8.3%).

The two methods of assessing sagittal plane alignment had the highest concordance in the 9 mm category (73.3%) and decreased with increasing insert size, as seen in the 10 mm (56.7%) and 11 mm (53.3%) categories. In particular, with both 10 and 11 mm inserts, the differences in distribution between the two assessment methods were significant, and the mismatch rate was highest at 46.7% with the 11 mm insert (*p* < 0.0001, *p* = 0.001).

### Inter-Observer Reliability (Secondary Analysis)

The inter-observer agreement for sagittal alignment assessment between the two orthopedic surgeons was classified as good (κ = 0.717, 95% CI: 0.65–0.80)

For continuous angle values, the ICC was 0.816 (95% CI: 0.74–0.87), indicating excellent reliability.

## 4. Discussion

In general, the angle formed by femoral and tibial mechanical axes in the sagittal plane is defined as hyperextension when <0° and as flexion contracture when >0° [11,12].

Genu recurvatum following TKA is rare (0.5–1%), but may contribute to joint instability, decreased function, recurrence, and the need for further intervention [4,13,14]. Conversely, a flexion contracture of greater than 5° can lead to gait disturbance, pain, and patient dissatisfaction, whereas a flexion contracture of greater than 15° is considered a major source of disability [5,15,16].

The mechanical axis of the femur in the sagittal plane is defined as the line connecting the center of the femoral head and the center of the condyles, while the mechanical axis of the tibia in the sagittal plane is defined as the line connecting the center of the proximal tibia and the center of the talus. However, due to the presence of surrounding thigh muscles, these anatomical landmarks cannot always be clearly identified intraoperatively. Seo et al. [17] therefore proposed defining the palpable mechanical axis as the line connecting the anterior margin of the greater trochanter and the center of the lateral epicondyle of the femur. They reported that the axis of the distal femoral anterior cortex in the sagittal plane was, on average, 4.1° (range, 1.5 to 11.7°; SD, 2.8°) more flexed than the sagittal mechanical axis and that the palpable mechanical axis was 2.4° (range, 0.4 to 4.2°; SD, 0.9°) more flexed than the sagittal mechanical axis. They found that palpable mechanical axis can be used to estimate sagittal plane alignment. However, the use of a proximal thigh tourniquet can make palpation of the greater trochanter difficult, leading to challenges assessing the mechanical axis intraoperatively [18].

In the present study, we also found that increased BMI > 25 kg/m^2^ and increased thigh circumference significantly interfered with intraoperative assessment of sagittal alignment (both *p* < 0.0001). This suggests that excess subcutaneous fat and limited visual exposure may obscure anatomical landmarks and lead to perceptual errors in estimation of the palpable mechanical axis.

By contrast, the MAKO robotic system utilizes the preoperative CT scan and anatomic landmarks based on intraoperative registration to recreate a 3-dimensional model to assess the extension angle [19,20]; therefore, its accuracy is less affected by the patient’s body habitus.

Previous studies have similarly reported that obesity can make anatomical landmark identification difficult during conventional TKA, contributing to errors in postoperative alignment [21]. In contrast, robotic- or navigation-assisted TKA maintains consistent alignment outcomes even in obese patients [22].

In our study, the largest discordance between surgeon’s visual assessment and MAKO measurements of hyperextension occurred with the 9 mm insert. This may be explained by biomechanical studies showing that thinner inserts increase ligamentous laxity and posterior femoral laxity in full extension [23]. Therefore, the tibia may appear anteriorly tilted, leading the surgeon to mistakenly perceive the knee to be in extension, when the joint is in mild flexion.

Furthermore, since the distal femoral anterior cortex is approximately 4.1° more flexed than the true sagittal mechanical axis and 2.4° more flexed than the palpable mechanical axis [17], relying on the palpable mechanical axis intraoperatively may cause the surgeon to misinterpret flexion as neutral or even hyperextension. Jacobs et al. also reported that surgeon’s judgment in the operating room tends to underestimate actual flexion contracture angles, leading to misclassification as hyperextension [18].

When agreement in sagittal alignment assessment between the two groups was compared according to the degree of flexion contracture, the opposite trend was observed when using the simple Neutral/FC/HE classification, and thicker inserts were associated with a higher rate of mismatch such that agreement on the presence of flexion contracture increased, but agreement on its severity decreased.

The MAKO system provides continuous, objective angle measurements, whereas surgeons typically rely on categorical judgments, such as “looks extended” or “appears flexed,” rather than precise angular measurements. When applying a ≥5° threshold to classify flexion contracture, the S group tended to overlook progressive increases with thicker inserts, categorizing them within the same range. Consequently, this resulted in mismatch with the continuous assessments of the M group. Therefore, relying solely on surgeon’s visual assessment may lead to underestimation of the flexion contracture.

In summary, in comparing sagittal plane hyperextension assessment between the surgeon’s visual evaluation and the MAKO robotic system during TKA, the S group showed a tendency to underestimate the mechanical axis in the sagittal plane among overweight/obese patients (BMI ≥ 25 kg/m^2^) (*p* < 0.001). In contrast, the M group demonstrated no statistically significant difference compared to radiographic measurements (*p* = 0.095). Visually, surgeons tended to overestimate hyperextension when using thinner trial inserts, while the severity of flexion contracture was underestimated when thicker inserts (11 mm) were used.

Therefore, several practical considerations can be suggested. First, during early trial stages with thinner inserts, an appearance of hyperextension should not immediately prompt an increase in insert thickness. Instead, concurrent confirmation with the robotic system’s assessment of sagittal plane alignment is recommended. Second, if the robotic system persistently indicates flexion contracture, unnecessary upsizing of the insert should be avoided. Instead, soft-tissue and bony adjustments such as posterior capsular release, debridement of posterior osteophytes, or re-evaluation of distal femoral and proximal tibial resections should be prioritized. Third, before selecting the final insert, it may be useful to record robotic alignment values for each insert thickness (9 → 10 → 11 mm) and perform additional balancing in areas where there is the largest discordance between visual assessment and robotic analysis. This can minimize the risk of instability or residual contracture caused by overcorrection or undercorrection.

In this study, sagittal alignment was independently evaluated by two orthopedic surgeons, and inter-observer reliability was reported quantitatively.

Agreement between the two evaluators was κ = 0.717 and ICC = 0.816, indicating reliability ranging from “good to excellent.” These results confirm that visual assessment by experienced orthopedic surgeons is consistently reproducible.

Nevertheless, the primary analysis was based on a single surgeon’s assessment, with inter-observer reliability reported as a secondary analysis. Accordingly, the primary outcome is limited by its reliance on a single evaluator’s assessment.

In circumstances where patient body habitus limits anatomical visualization or when subtle variations in ligament tension must be carefully assessed, relying solely on the surgeon’s visual assessment may necessitate more cautious decisions regarding insert thickness. In such cases, quantitative alignment assessment with a robotic-assisted system provides a valuable adjunct, functioning as a key tool to ensure more consistent and reproducible surgical outcomes.

The strengths of this study include the use of objective robotic measurements validated by intraoperative fluoroscopy and the evaluation of multiple anatomical factors influencing visual accuracy and the novel focus on sagittal alignment evaluation in robotic TKA, which has not been thoroughly investigated in previous studies. However, as a retrospective single-center study with limited sample size, potential selection bias and lack of postoperative functional outcomes should be acknowledged. Further large-scale studies with long-term follow-up are warranted to validate these findings and optimize surgical strategies for improved patient satisfaction and functional results.

## Figures and Tables

**Table 1:** Demographics of a normal-weight group and an overweight/obesity group.

	Normal-Weight Group (BMI < 25 kg/m^2^)	Overweight/Obesity Group (BMI ≥ 25 kg/m^2^)	*p*-Value
Number of cases	28	32	
Age (years)	73.1 (±5.8)	72.4 (±8.0)	0.676
BMI (kg/m^2^)	22.9 (±1.3)	28.8 (±1.6)	<0.0001
Preoperative HKA (°)	8.1 (±3.3)	8.5 (±3.4)	0.668
Preoperative tibia/femur length ratio	0.4 (±0.2)	0.4 (±0.2)	0.186
Preoperative height/lower extremity length ratio	0.2 (±0.0)	0.2 (±0.0)	0.182
Thigh circumference (cm)	45.4 (±3.3)	48.5 (±4.2)	0.002

Data are presented as mean ± SD unless otherwise indicated. BMI: Body mass index, HKA: Hip–knee–ankle.

**Table 2:** Comparison of sagittal plane alignment angles assessed by fluoroscopy, surgeon’s visual estimation, and MAKO robotic system in normal-weight and overweight/obesity groups.

	Normal-Weight Group	*p*-Value	Overweight/Obesity Group	*p*-Value
C-arm (°)	3.4 (±3.4)	0.095	2.6 (±2.7)	<0.0001
Surgeon’s eyes (°)	2.0 (±2.7)		0.1 (±2.7)	
C-arm (°)	3.4 (±3.4)	0.437	2.6 (±2.7)	0.607
MAKO (°)	2.7 (±3.8)		3.0 (±3.0)	

Data are presented as mean ± SD unless otherwise indicated.

**Table 3:** Comparison of sagittal alignment assessment between surgeon’s visual estimation and MAKO robotic system based on insert thickness (9 mm, 10 mm, 11 mm). categorized into neutral (0°), flexion contracture (>0°), and hyperextension (<0°).

Insert Thickness	Alignment (°)	Surgeon’s Eyes, *n* (%)	MAKO, *n* (%)	Match:Mismatch (%, *n*)	*p*-Value
9 mm	0°	34 (56.7)	7 (11.7)	27:33 (45.0:55.0)	<0.0001
	>0° (FC)	7 (11.7)	35 (58.3)		
	<0° (HE)	19 (31.7)	18 (30.0)		
10 mm	0°	12 (20.0)	10 (16.7)	38:22 (63.3:36.7)	0.05
	>0° (FC)	34 (56.7)	45 (75.0)		
	<0° (HE)	14 (23.3)	5 (8.3)		
11 mm	0°	12 (20.0)	0 (0.0)	45:15 (75.0:25.0)	<0.0001
	>0° (FC)	42 (70.0)	55 (91.7)		
	<0° (HE)	6 (10.0)	5 (8.3)		

FC: Flexion Contracture; HE: Hyperextension.

**Table 4:** Comparison of flexion contracture and hyperextension severity between surgeon’s visual assessment and MAKO robotic system across insert thickness levels (9 mm, 10 mm, 11 mm). Flexion contracture was subcategorized into <5° and ≥5°, and hyperextension was defined as <0°.

Insert (Stratum)	Category	Surgeon’s Eyes, *n* (%)	MAKO, *n* (%)	Match:Mismatch (*n*, %)	*p*-Value
9 mm	<5° flexion	41 (68.3)	37 (61.7)	44:16 (73.3:26.7)	0.098
	≥5° flexion	0 (0.0)	5 (8.3)		
	<0° hyperextension	19 (31.7)	18 (30.0)		
10 mm	<5° flexion	45 (75.0)	37 (61.7)	34:26 (56.7:43.3)	<0.0001
	≥5° flexion	1 (1.1)	18 (30.0)		
	<0° hyperextension	14 (23.3)	5 (8.3)		
11 mm	<5° flexion	37 (61.7)	18 (30.0)	32:28 (53.3:46.7)	0.001
	≥5° flexion	17 (28.3)	37 (61.7)		
	<0° hyperextension	6 (10.0)	5 (8.3)		

## Data Availability

The data presented in this study are available upon reasonable request from the corresponding author.

## Abbreviations

The following abbreviations are used in this manuscript:

TKA	Total Knee Arthroplasty
BMI	Body Mass Index
ICC	Intra-class Correlation Coefficient
SD	Standard Deviation
HKA	Hip–Knee–Ankle
